# Up-Regulation of Hepatitis C Virus Replication and Production by Inhibition of MEK/ERK Signaling

**DOI:** 10.1371/journal.pone.0007498

**Published:** 2009-10-16

**Authors:** Jean Ndjomou, In-woo Park, Ying Liu, Lindsey D. Mayo, Johnny J. He

**Affiliations:** 1 Department of Microbiology and Immunology, Indiana University School of Medicine, Indianapolis, Indiana, United States of America; 2 Department of Pediatrics, Indiana University School of Medicine, Indianapolis, Indiana, United States of America; 3 Center for AIDS Research, Indiana University School of Medicine, Indianapolis, Indiana, United States of America; Pohang University of Science and Technology, Republic of Korea

## Abstract

**Background:**

Viruses interact with and exploit the host cellular machinery for their multiplication and propagation. The MEK/ERK signaling pathway positively regulates replication of many RNA viruses. However, whether and how this signaling pathway affects hepatitis C virus (HCV) replication and production is not well understood.

**Methods and Results:**

In this study, we took advantage of two well-characterized MEK/ERK inhibitors and MEK/ERK dominant negative mutants and investigated the roles of the MEK/ERK signaling pathway in HCV gene expression and replication. We showed that inhibition of MEK/ERK signaling enhanced HCV gene expression, plus- and minus-strand RNA synthesis, and virus production. In addition, we showed that this enhancement was independent of interferon-α (IFN-α) antiviral activity and did not require prior activation of the MEK/ERK signaling pathway. Furthermore, we showed that only MEK and ERK-2 but not ERK-1 was involved in HCV replication, likely through regulation of HCV RNA translation.

**Conclusions:**

Taken together, these results demonstrate a negative regulatory role of the MEK/ERK signaling pathway in HCV replication and suggest a potential risk in targeting this signaling pathway to treat and prevent neoplastic transformation of HCV-infected liver cells.

## Introduction

Hepatitis C virus (HCV) causes chronic hepatitis, cirrhosis, and hepatocellular carcinoma (HCC) [1]. There are approximately 170 million infected people worldwide [2] and no therapeutic or prophylactic vaccines currently available. Therefore, HCV continues to represent a significant public health problem that mandates intensified efforts and investment in both clinical management and research.

HCV belongs to the *hepacivirus* genus in the *flaviviridae* family. It is a plus-strand RNA virus with a genome of approximate 9.6 kb. It contains a single open reading frame (ORF) encoding a single polyprotein precursor of 3,010 amino acids. The ORF is flanked by 5′ and 3′ untranslated regions (UTR). Viral proteins are translated through an internal ribosome entry site (IRES)-dependent mechanism. The precursor polyprotein is processed co- and post-translationally by cellular and viral proteases into 10 proteins: structural proteins (core, E1, and E2), a small membrane-associated protein p7, and six nonstructural (NS) proteins NS2, 3, 4A, 4B, 5A, and 5B [3].

The outcomes of HCV infection vary among individuals. Only a few percentage of the infected individuals are able to clear and resolve the infection, the remaining majority (50–80%) develop chronic hepatitis and other liver complications [2]. The viral mechanisms of chronic infection and the cellular determinants of infection clearance are poorly understood. Many viruses including HCV have developed sophisticated mechanisms to evade or antagonize cellular anti-viral responses, leading to persistent and/or chronic infection. The Raf/MEK/ERK signaling pathway is one of the mitogen-activated protein kinase (MAPK) cascades and plays important roles in the regulation of cell growth, differentiation, survival, and transmission of oncogenic signals [4]. This pathway is activated by a variety of stimuli including growth factors, mitogens, transforming agents, and virus infections. Upon stimulation, activated Raf kinase activates MEK1/2, which in turn activates ERK1/2 to phosphorylate substrates in the cytoplasm or to translocate to the nucleus to phosphorylate transcription factors and regulate target gene expression. MAPK also phosphorylates and activates other kinases termed MAPK-activated protein kinases including the 90-kDa ribosomal S6 kinases, the mitogen-and stress-activated kinases, and the MAPK-interacting kinases [5].

Viruses are intracellular obligate parasites; they have evolved to exploit the host cellular machinery for their replication. The MEK/ERK signaling pathway plays an important role in their replication. Activation of the MEK/ERK signaling cascade enhances replication of viruses such as human immunodeficiency virus [6], influenza virus [7], borna disease virus [8], coronavirus [9], coxsakievirus B3 [10], and herpes simples virus [11]. The underlying molecular mechanisms include interference with viral translational and replication machineries as well as host anti-viral defense. On the other hand, activation of the MEK/ERK signaling leads to inhibitory effects of virus replication as in the case of hepatitis B virus, a hepatotropic virus which like HCV induces hepatocellular carcinoma in infected hosts [12]. Meanwhile, interleukin-1 (IL-1) inhibits HCV replication in HCV subgenomic replicon cells through activation of ERK and induction of interferon (IFN)-stimulated gene 1-8U; MEK inhibitor PD98059 abrogates the inhibitory effects of IL-1 on HCV replication [13]. IL-1 is also involved in IFN-α-mediated antiviral gene induction in human hepatoma cells [14]. Taken together, these findings indicate that IL-1-mediated ERK activation induces an anti-viral effect. As the nature of the stimuli determines the final outcome of the signaling, it is conceivable that other stimuli of ERK signaling may have different effects on HCV replication. Moreover, it is not known whether and how the MEK/ERK signaling affects HCV replication. Therefore, in this study we aimed to characterize the role of this signaling pathway in HCV replication and production using both HCV subgenomic replicon and full-length genomic systems.

## Materials and Methods

### Cell culture and reagents

Huh 7-based HCV subgenomic replicon cells carrying a *Renilla* luciferase (RLuc) reporter were described elsewhere [15] and maintained in DMEM supplemented with 10% FBS, 1% penicillin/streptomycin/L-glutamine, 1X non-essential amino-acids, and 250 µg/ml G418. Huh 7 cells were a gift from Dr. Steve Polyak of University of Washington, Seattle; Huh 7.5 cells were obtained from Dr. Charles Rice of Rockefeller University, New York. Both cells were maintained as Huh 7 HCV replicon cells except for that no G418 was present in the culture medium. ERK-1 mAb was from BD Biosciences (San Jose, CA), MEK1/2 mAb from Cell Signaling Technology (Danvers, MA), HCV core mAb from Affinity Bioreagents (Golden, CO), HA antibody from Santa Cruz Biotechnologies (Santa Cruz, CA), and β-actin mAb from Sigma (Saint Louis, MO). MEK inhibitors U0126 and PD98059 were from Cell Signaling Technology and were reconstituted in DMSO. Interferon-α/2b (IFN-α/2b) was purchased from PBL Biomedical Laboratories (Piscataway, NJ) and reconstituted in PBS.

### Plasmids

pCR2.1-β-actin was constructed by PCR-cloning of β-actin fragment into pCR2.1-TOPO vector (Invitrogen, Carlsbad, CA). pFL-JFH1 was obtained from Dr. Takaji Wakita [16]. Dominant negative MEK-1, ERK-1, and ERK-2 were described elsewhere [17]. pGEM-3Z-5′UTR was previously constructed [18]. pC3.RLuc.HCV IRES.FLuc was constructed from pC3.RLuc.Polio IRES.Fluc, a gift from Dr. Nahum Sonenberg of McGill University, Canada by replacing the polio IRES fragment with the HCV IRES at Kpn I and BamH I restriction sites.

### *In vitro* RNA transcription

HCV RLuc replicon construct was linearized with Sca I, purified, and transcribed with MEGAscript™ T7 RNA transcription kit (Ambion, Austin, TX). The RNA transcripts were treated with 2 U DNase I at 37°C for 30 min, purified by acid phenol chloroform extraction, and suspended in diethylpyrocarbonate-treated water. To synthesize the full-length HCV JFH-1 RNA, the pFL-JFH1 plasmid was linearized with Xba I, purified, and used as template for *in vitro* transcription as above. Transcribed RNA was stored in aliquots at −80°C.

### Transfection of HCV RNA, plasmids, and siRNA

Cells were plated in a 24-well plate at a density of 8×10^4^ cells per well or in a 6-well plate at a density of 8×10^5^ cells per well. Transfection of HCV RNA and dominant negative constructs were performed using Lipofectamine 2000 reagents (Invitrogen) according to the manufacturer's instructions. ERK-1-specific siRNA and control siRNA were purchased from Ambion and transfected into the cells using Oligofectamine (Invitrogen) following the manufacturer's instructions. In all transfections, the cells were washed by and replaced with the fresh medium 24 hr after transfection.

### Immunoblotting

Cell lysates were prepared at 48 hr post transfection with a standard RIPA buffer and separated on a 12% polyacrylamide-SDS gel. The proteins were transferred onto the nitrocellulose membrane, probed with appropriate primary antibodies and HRP-conjugated secondary antibodies, and were visualized by the enhanced chemiluminescence (VWR, West Chester, PA). As indicated, membrane was stripped and re-probed with anti-β-actin mAb.

### Luciferase reporter gene assay

HCV RLuc replicon cells were grown in a 24-well plate and treated with MEK inhibitors or their solvent DMSO for 24 hr or pre-treated with IFN-α/2b or its solvent PBS prior to treatment with MEK inhibitors or DMSO. The luciferase assay was performed using a *Renilla* luciferase assay kit (Promega, Madison, WI) according to the manufacturer's instructions. In experiments involving dominant negative constructs and siRNA, HCV RLuc replicon cells were transfected with these plasmids or siRNA, cultured for 48 hr, and then harvested for the luciferase assay. All luciferase assays were done in triplicates and the results were expressed as mean±SEM. The difference was considered statistically significant when the *p* value was less than 0.05.

### RNase protection assay (RPA)

HCV RLuc replicon cells were grown in a 6-well plate, treated with MEK inhibitors and/or IFN-α/2b or transfected with siRNAs as indicated. Total RNA was isolated using TRIzol®Reagent (Invitrogen). β-actin probe and HCV sense and anti-sense 5′UTR probes were synthesized using pCR2.1-β-actin and pGEM-3Z-5′UTR as respective templates and a Maxiscript™ RNA transcription kit (Ambion) according to the manufacturer's instructions. The RPA was performed with a BD RiboQuant™ ribonuclease protection assay kit (BD Biosciences) as described elsewhere [18]. Briefly, 10 µg total RNA was hybridized with 80,000 cpm [α-^32^P] UTP-labeled 5′UTR anti-sense or sense probe for detection of plus- or minus-strand HCV RNA respectively. The β-actin RNA was used as an internal equal RNA loading control and detected with 5,000 cpm [α-^32^P] UTP-labeled β-actin anti-sense probe. The protected probes were analyzed on 6% polyacrylamide-7.5 M urea gel and visualized by autoradiography.

### Quantitative real-time RT-PCR for HCV RNA

The culture supernatant from Huh 7.5 cells transfected with HCV JFH-1 RNA was collected, filtered through 0.22 µm filter, and concentrated by about 30 times using an Amicon® Ultra-15 Centrifugal Filter Unit (MW cut-off 100 kDa) (Millipore, Bedford, MA). The RNA was isolated from the concentrated supernatant using a QIAamp® Viral RNA Mini Kit (Qiagen, Valencia, CA) to determine HCV production in the cell culture supernatant. Intracellular HCV RNA was also isolated from these transfected cells. Both RNA were quantitated in triplicates using a TaqMan® OneStep RT-PCR Master Mix Reagents Kit (Applied Biosystems, Foster City, CA) and the primers and probes described previously [19]. The reactions were performed on a Mx3000P QPCR system (Stratagene, La Jolla, CA) with a program of 48°C for 30 min, 95°C for 10 min, and then 50 cycles at 95°C for 15 sec and 60°C for 1 min. The absolute HCV RNA level was calculated using *in vitro* transcribed JFH-1 RNA standards.

## Results

### MEK inhibitors enhanced HCV replication

**Figure 1:**
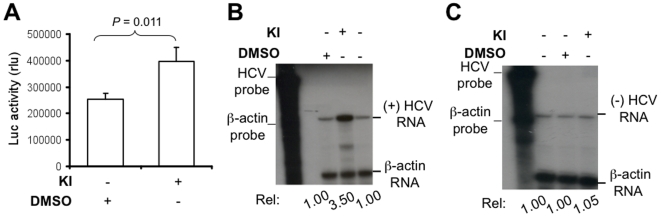
MEK inhibitors enhanced HCV protein and RNA syntheses. HCV RLuc replicon cells were grown in a 24- or 6-well plate and treated with either DMSO or MEK inhibitors U0126 (5 µM) and PD98059 (10 µM) for 24 hr. A. Lysates were subjected to the luciferase reporter gene activity assay and data were expressed as mean±SEM of triplicates. KI: both MEK inhibitors. Total RNA was used for the detection of plus-strand HCV RNA using the anti-sense RNA probe (B) and minus-strand HCV RNA using the sense RNA probe (C) by RPA. β-actin RNA was detected using a β-actin probe to ensure loading of equal amount of RNA. The levels of plus- and minus-strand HCV RNA were determined by densitometry relative to that of the DMSO control. The data shown are representative of three independent experiments.

To determine the relationship between MEK/ERK signaling and HCV replication, we took advantage of two well-characterized and widely used MEK inhibitors (U0126 and PD98059). U0126 inhibits the active and inactive forms of MEK1/2, while PD98059 inhibits the activation of inactive MEK1/2 [20], . In the preliminary experiments, we titrated the concentrations of U0126 and PD98059 for a maximal MEK/ERK inhibitory effect while with little cytotoxicity and confirmed the optimal concentrations for U0126 and PD98059 to be 5 µM and 10 µM, respectively for their combined use (data not shown). We then used these concentrations to examine the effects of these inhibitors on HCV replication in HCV RLuc replicon cells. Compared to the DMSO control, treatment of MEK inhibitors resulted in a significant increase in the luciferase reporter gene activity (Fig. 1A). We next determined whether these inhibitors affected HCV RNA synthesis. To distinguish between plus- and minus-strand HCV RNA syntheses, we performed the RNase protection assay using the strand-specific probes. Before using this assay, we tested the strand specificity on *in vitro* transcribed HCV 5′ UTR RNA of plus- and minus-polarities by hybridization with the anti-sense and sense probes, we found that the sense probe only detected the minus-strand RNA and the anti-sense probe only detected the plus-strand RNA and that there was no cross by hybridization (data not shown). Treatment of the inhibitors increased plus-strand HCV RNA level by about 3.5 fold over the DMSO control (Fig. 1B) and had only a slight increase of minus-strand HCV RNA level over the DMSO control (Fig. 1C). Taken together, these results provide the first evidence that inhibition of the MEK/ERK signaling enhanced HCV gene expression and replication.

### Enhancement of HCV replication by MEK inhibitors was independent of IFN-α anti-viral activity

**Figure 2:**
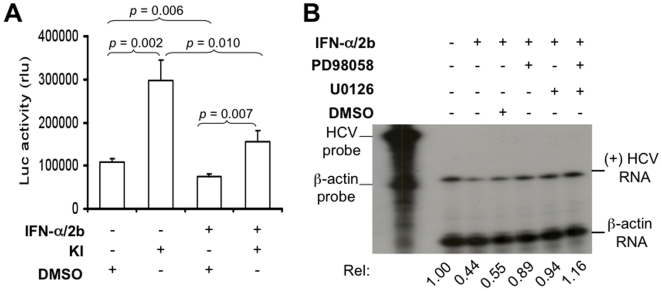
MEK inhibitors-enhanceed HCV replication was independent of the IFN-α anti-viral activity. HCV replicon cells were grown in a 24- or 6-well plate in the presence of IFN-α/2b (200 IU/ml) for 24 hr and then treated with either MEK inhibitors or DMSO for 24 hr. A. Lysates were subjected to the luciferase reporter gene assay and the data were expressed as mean±SEM of triplicates. B. Total RNA was assayed for plus-strand HCV RNA by RPA and the data were quantitated by densitometry as above.

HCV replication is inhibited by IFN-α treatment [22]. IFN-α binds to its cognate receptors and activates the JAK/STAT signaling, which in turn induces effectors such as MxA guanosine triphosphatase, dsRNA activated-protein kinase, and 2′-5′oligoadenylate synthase and accounts for IFN-α-mediated anti-viral activity [23]. To investigate whether MEK inhibitors enhanced HCV replication by antagonizing the well-characterized anti-viral mechanism of IFN-α, we treated HCV RLuc replicon cells first with IFN-α/2b, followed by MEK inhibitors. Lysates were prepared for the luciferase reporter assay. As expected, IFN-α/2b treatment alone inhibited the luciferase reporter gene activity (*p* = 0.006). Treatment with IFN-α/2b followed by MEK inhibitors showed a significantly higher luciferase activity compared to IFN-α treatment control alone (*p* = 0.007), but it was significantly lower than the MEK inhibitors alone (*p* = 0.010) (Fig. 2A). We also determined HCV RNA replication in these cells. We treated the cells as above, isolated total RNA, and performed RPA to detect plus-strand HCV RNA. In agreement with the luciferase reporter gene activity, IFN-α/2b inhibited plus-strand HCV RNA synthesis (lane 3, Fig. 2B) when compared to its PBS control (lane 2, Fig. 2B); IFN-α/2b followed by each or both MEK inhibitors increased plus-strand HCV RNA synthesis (lane5, 6 & 7, Fig. 2B) when compared to the IFN-α/2b plus DMSO control (lane 4, Fig. 2B). In addition, the effect of both inhibitors on plus-strand HCV RNA synthesis was higher than each inhibitor alone (Fig. 2B). These findings suggest that enhanced HCV replication by MEK inhibitors is probably independent of the IFN-α anti-viral activity.

### The basal activity of MEK was sufficient for MEK inhibitors-enhanced HCV replication

**Figure 3:**
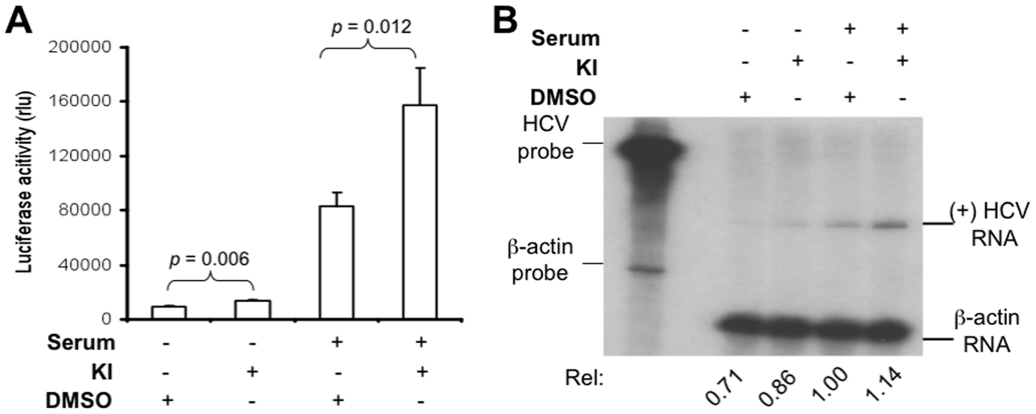
The basal activity of MEK/ERK signaling was sufficient for MEK inhibitors-enhanced HCV replication. HCV replicon cells were cultured in a 24- or 6-well plate in the absence or presence of serum for 24 hr and treated with either MEK inhibitors or DMSO for 24 hr. A. Lysates were subjected to the luciferase reporter gene activity assay and the data were expressed as mean±SEM of triplicates. B. Total RNA was assayed for plus-strand HCV RNA by RPA and the data were quantitated as above and is representative of three independent experiments.

The above experiments were conducted with cells that were cultured in the medium containing 10% FBS, which likely activated the basal MEK/ERK signaling. Thus, we then determined whether the basal level of the MEK/ERK activity was sufficient for the MEK inhibitors-enhanced HCV replication. To address this issue, we cultured HCV RLuc replicon cells in the serum-free medium for 24 hr and then treated with MEK inhibitors. Lysates were prepared for the luciferase reporter activity assay, and total RNA was isolated for plus-strand HCV RNA detection by RPA. In the absence of serum in the culture medium, MEK inhibitors also significantly increased the luciferase reporter gene activity when compared to the DMSO control (*p* = 0.006) (Fig. 3A). In parallel, MEK inhibitors also increased the plus-strand HCV RNA level (Fig. 3B). However, it is very interesting to note that the overall luciferase activity of the cells cultured in the 10%-FBS-containing medium was considerably higher than that of the cells cultured in serum-free medium, despite that the enhancement effects by these inhibitors showed little difference between cells cultured in the presence of serum and in the absence of serum (Fig. 3A). This can be attributed to activation of the phosphatidylinositol 3-kinase (PI3K)/Akt pathway, which is the other major signaling pathway involved in serum-dependent cell survival and growth. Taken together, these results suggest that prior activation of MEK/ERK signaling is not necessary for MEK inhibitors-enhanced HCV replication, but it is involved in regulation of HCV replication.

### Dominant negative MEK-1 and ERK-2 but not ERK-1 enhanced HCV replication

**Figure 4:**
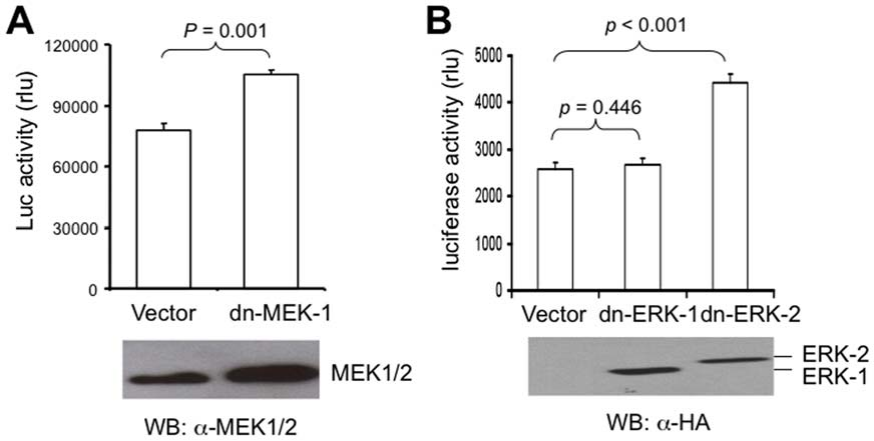
Dominant negatives MEK-1 and ERK-2 but not ERK-1 up-regulated HCV replication. HCV RLuc replicon cells were transfected with dominant negative MEK-1 (A), ERK-1 or ERK-2 (B), or the empty vector. At 48 hr post-transfection, lysates were subjected to Western blot analysis or the luciferase reporter gene activity assay. The luciferase activity was normalized to the same number of cells. The data were expressed as mean±SEM of triplicates.

To ensure that MEK inhibitors-enhanced HCV replication was not due to a non-specific off-target effect of these drugs and to test whether the responsible effectors were downstream of MEK, we took advantage of the dominant negative strategy to inactivate the constitutive MEK-1, ERK-1, and ERK-2 activity and determined their effects on HCV replication. HCV RLuc replicon cells were transfected with dominant negative MEK-1, ERK-1, ERK-2 constructs [17] or an empty vector control. Lysates were prepared at 48 hr post-transfection and the luciferase gene activity was measured. Expression of these dominant negatives was detected by Western blot analysis (bottom panels, Fig. 4A&B). Expression of dominant negative MEK-1 led to a significantly higher luciferase reporter gene activity compared to that of the control (*p* = 0.001) (Fig. 4A). Similarly, expression of ERK-2 dominant negative significantly increased the luciferase reporter gene activity (*p*<0.001) (Fig. 4B). However, expression of dominant negative ERK-1 had little effect on the luciferase gene activity compared to the control (*p* = 0.445) (Fig. 4B). Theses results are in agreement with those obtained with MEK inhibitors and also support the role of MEK-1 and ERK-2 but not ERK-1 in HCV replication.

### ERK-1 knockdown had no effects on HCV replication

**Figure 5:**
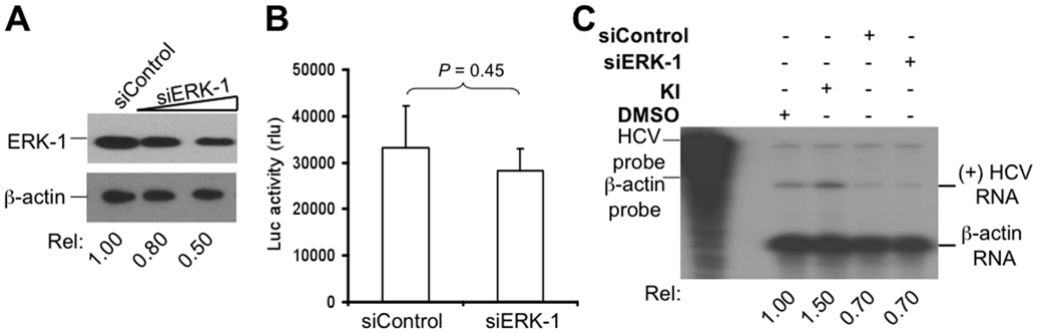
ERK-1 knockdown had no effects on HCV replication. A. HCV replicon cells were transfected with siERK-1 (50 nM and 300 nM) or unrelated control siRNA. Forty-eight hours post-transfection, lysates were subjected to Western blot analysis using anti-ERK-1 mAb. Blot was re-probed with anti-β-actin mAb to ensure equal protein loading. HCV replicon cells were grown in a 24- or 6-well plate and transfected with siRNAs as above. B. Lysates were used for the luciferase reporter gene activity assay. The data were expressed as mean±SEM of triplicates. C. Total RNA was used for the detection of plus-strand HCV RNA by RPA using a anti-sense HCV probe. β-Actin RNA was detected as internal equal RNA loading control using a β-actin-specific probe. RNA bands were quantified by densitometry, normalized to the β-actin internal control, and expressed as fold induction relative to the DMSO control.

To ascertain that ERK-1 was not involved in HCV replication, we used the siRNA strategy to knock down the constitutive ERK-1 expression and determined its effects on HCV replication. HCV RLuc replicon cells were transfected with ERK-1-specific siRNA or the control siRNA. Lysates were prepared for ERK-1 expression by Western blot analysis and the effect of ERK-1 silencing on HCV was assessed by measuring the luciferase gene activity. ERK-1 siRNA effectively knocked down the constitutive ERK-1 expression (Fig. 5A). However, ERK-1 knockdown did not exhibit a significant difference in the luciferase gene activity compared to the control siRNA (*p* = 0.45) (Fig. 5B). Both control and ERK-1 siRNA appeared to have non-specific effects on the level of plus-strand HCV RNA synthesis, but there was no difference between these two transfections (Fig. 5C). Therefore, these results further support the notion that ERK-1 does not play any significant roles in HCV replication.

### MEK inhibitors enhanced HCV replication and production in a full-length HCV genomic system

**Figure 6:**
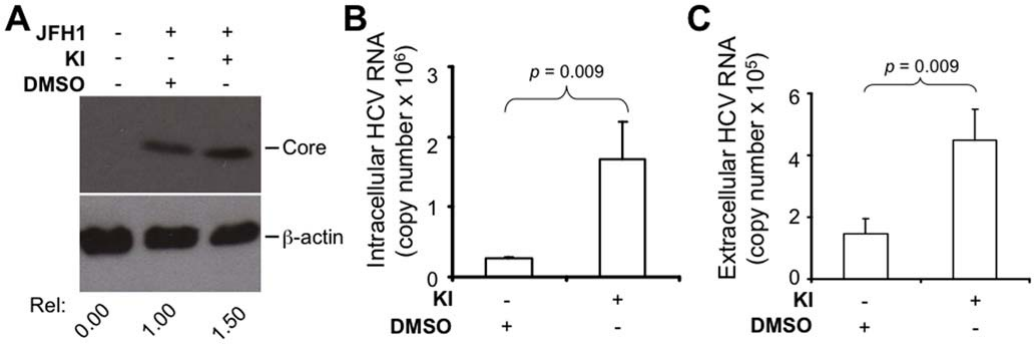
MEK inhibitors enhanced HCV gene expression and virus production. Huh 7.5 cells were transfected with full-length JFH-1 RNA and treated with either DMSO or MEK inhibitors for 48 hr. A. Lysates were analyzed for HCV core by Western blot using anti-core mAb and the blot was re-probed for β-actin for equal protein loading. B. Total RNA was extracted and intracellular HCV RNA was quantitated by real-time RT-PCR using the TaqMan technology. C. Supernatants were collected, concentrated, and viral RNA was isolated with Qiagen viral RNA kit. HCV RNA in the supernatants was quantitated as above. Serially diluted *in vitro* transcribed HCV RNAs were used as standards for real-time RT-PCR quantitation. The data were expressed as mean±SEM of triplicates and were representative of three independent experiments.

Having found that MEK inhibitors increased HCV replication in the subgenomic replicon cells harboring HCV genotype 1b, we then wished to determine whether these inhibitors also increased HCV gene expression, replication and production in other genotypes. We addressed these questions using the recently characterized infectious genotype 2a HCV strain JFH-1 [16]. We transfected Huh 7.5 cells with *in vitro* transcribed full-length JFH-1 RNA and then treated the cells with MEK inhibitors or the control DMSO. Western blot analysis of cell lysates showed that treatment of MEK inhibitors resulted in higher HCV core protein expression compared to the DMSO control (Fig. 6A). Analysis of total cellular RNA by real-time RT-PCR showed that treatment of MEK inhibitors also led to a significantly higher level of plus-strand HCV RNA compared to the DMSO control (*p* = 0.009) (Fig. 6B). We next determined whether the intracellular increase in HCV proteins and RNA led to more extracellular virus production. To do this, we collected supernatants from JFH-1 RNA-transfected Huh7.5 cells that were treated with MEK inhibitors or DMSO control, isolated the RNA from the supernatants and quantified HCV RNA in the supernatants by real-time RT-PCR. In agreement with the intracellular increases in HCV protein and RNA syntheses, treatment of MEK inhibitors led to a significantly higher level of extracellular HCV production compared to the DMSO control (*p* = 0.009) (Fig. 6C). Taken together, these results indicate that inhibition of MEK signaling increases HCV gene expression and production in a full-length HCV genomic system, therefore pointing to a general mechanism by which the MEK/ERK signaling controls HCV replication.

### MEK inhibitors enhanced HCV IRES-dependent but not cap-dependent translation

**Figure 7:**
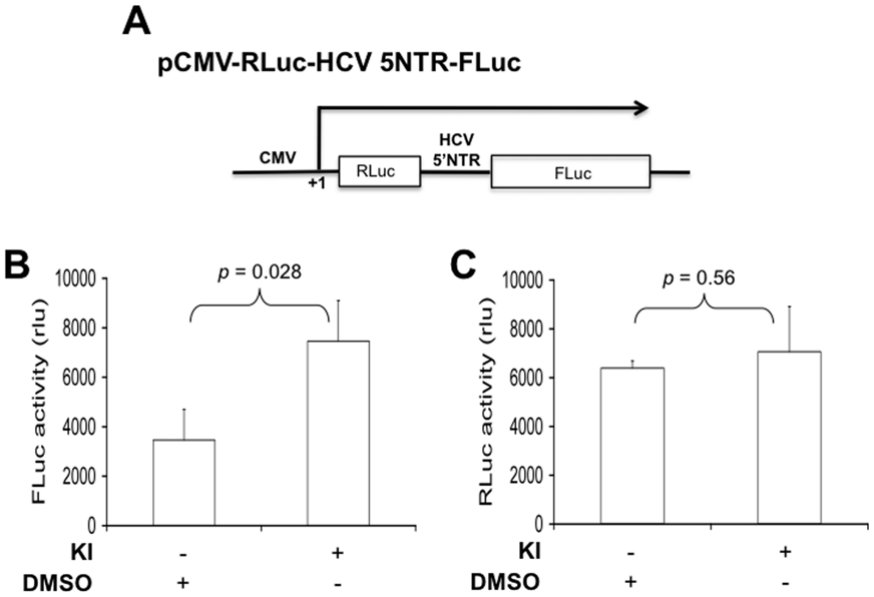
MEK inhibitors enhanced HCV IRES-dependent but not cap-dependent translation. Huh 7 cells were transfected with the bicistronic reporter construct pC3.RLuc.HCV.IRES.Fluc (A) and treated with either DMSO or MEK inhibitors for 24 hr. Lysates were assayed for the *Firefly* luciferase reporter gene activity to measure HCV IRES-dependent translational activity (B) and the *Renilla* luciferase reporter gene activity to measure cap-dependent translation (C). The data were expressed as means±SEM of triplicates and were representative of three independent experiments.

Besides regulating targets gene expression by phosphorylating downstream transcription factors, the MEK/ERK signaling cascade is also involved in the translational control of gene expression through direct phosphorylation of translation factors [5]. Thus, we also determined whether the MEK signaling was involved in regulation of HCV IRES-dependent translation. To test this hypothesis, we constructed a bicistronic reporter plasmid in which HCV IRES drives the translation of the *Firefly* luciferase gene while the *Renilla* luciferase gene is translated by a cap-dependent mechanism (Fig. 7A). We transfected Huh 7 cells with the bicistronic reporter, treated the cells with MEK inhibitors, and harvested cells for the *Renilla* luciferase (RLuc) and *Firefly* luciferase (FLuc) activity. Treatment of MEK inhibitors significantly increased *Firefly* luciferase reporter gene activity, i.e. the HCV IRES-dependent translation compared to the DMSO control (p = 0.028) (Fig. 7B). However, no statistically significant difference for the *Renilla* luciferase reporter gene activity, i.e. the cap-dependent translation was observed between cells treated with MEK inhibitors and DMSO (p = 0.56) (Fig. 7C). These results indicate that the MEK signaling is also involved in regulation of the HCV IRES translational activity.

## Discussion

In this study, we took advantage of MEK inhibitors and dominant negatives and demonstrated the involvement of MEK-1 and ERK-2 but not ERK-1 in HCV replication in both HCV subgenomic and genomic systems. We found that blockage of MEK/ERK signaling by MEK inhibitors increased HCV gene expression and plus-strand RNA synthesis, which is consistent with previous studies [13], [15], . In this study, we further looked at the effects of these inhibitors on HCV minus-strand RNA synthesis and found only a slight increase. This finding could be well explained by the well-known asymmetric nature of plus- and minus-strands HCV RNA synthesis in HCV-infected cells. HCV RNA replication starts with the synthesis of genome-length negative strand RNA which in turn serves as template for multiple rounds of nascent, positive-strand RNA synthesis, leading to an asymmetric accumulation of nearly 10 plus-strand RNA for every single minus-strand RNA [25]. These results imply that the MEK/ERK signaling pathway plays an important role in HCV replication. We further examined and ruled out the possibility that these inhibitors act off-target to produce the observed effects by the findings that dominant negative MEK-1 expression significantly enhanced HCV replication.

Previous studies have shown that disruption of ERK-dependent type I IFN induction breaks the myxoma virus species barrier [26]. Another study has also reported a negative regulation of IFN-α-induced anti-viral response to vesicular stomatitis virus and the Raf/MEK signaling pathway [27]. This led us to investigate whether antagonizing the IFN-α anti-viral activity was an alternative mechanism by which the MEK/ERK inhibitors produce the observed effects. However, we found no inverse correlation between the IFN-α anti-viral activity and MEK inhibitors-enhanced HCV replication, arguing against the possibility that these inhibitors or dominant negatives enhanced HCV replication by interfering with the IFN-α anti-viral response.

The MEK/ERK signaling pathway may regulate HCV replication through direct or indirect phosphorylation of viral and/or cellular proteins. In the case of viral proteins, for example, phosphorylation of NS5A and NS5B proteins has been shown to regulate HCV replication [28], [29]. Moreover, selective inhibition of cellular kinases has found an inverse correlation between NS5A phosphorylation level and HCV RNA replication [29]. In addition, NS5A protein is phosphophorylated on serine and threonine residues [30], [31] making it a potential substrate for MEK kinase. While one study has shown little change in the level of NS5A phosphorylation after treatment with the MEK kinase inhibitor PD98059 [24], the other one has demonstrated an inverse correlation between inhibition of MEK kinase, the level of NS5A phosphorylation and HCV replication [15]. However, our findings that dominant negative ERK-2 expression enhanced HCV replication clearly suggest that the effectors of MEK kinase-mediated control of HCV replication are located downstream of MEK kinase. Therefore, future investigation of the direct phosphorylation of NS5A by MEK would help determine whether the viral NS5A is a direct substrate for this cellular kinase.

The MEK/ERK signaling pathway is involved in cell growth and proliferation and has been a major target for anti-cancer drugs development. HCV induces hepatocellular carcinoma in infected individuals; our findings in this study as well as others [13], [15], [24] that inhibition of MEK signaling leads to the up-regulation of HCV replication raise concerns about the potential use of those inhibitors for the treatment of cancer in HCV-infected individuals. This led us to evaluate the outcome of MEK inhibitors-enhanced HCV replication on virus production using the recently described HCV JFH-1 cell culture system [16]. We found a positive correlation among the increase in HCV proteins, plus-strand RNA accumulation in the cells, and HCV production in the culture supernatant. These findings support the notion that targeting MEK/ERK signaling as therapeutic intervention to treat hepatocellular carcinoma may lead to acceleration of HCV pathogenic process. This possibility is further supported by an early report that treatment of cells with MEK kinase inhibitor PD98059 indeed increases the infectivity of HCV-positive serum [24].

The MEK/ERK signaling pathway regulates target gene expression at both the transcriptional and translational levels by direct phosphorylation of translation factors [5]. Using a bicistronic reporter system, we found that MEK inhibitors enhanced HCV IRES translational activity but had no effects on the cap-dependent translation. These findings are consistent with a previous report [24] and supports the notion that the MEK/ERK signaling functions to control HCV replication through the HCV translational machinery. Furthermore, these findings suggest that the HCV IRES translational machinery can be specifically targeted with pharmacological agents without affecting the host translational system.
